# Degradation of insulin amyloid by antibiotic minocycline and formation of toxic intermediates

**DOI:** 10.1038/s41598-021-86001-y

**Published:** 2021-03-25

**Authors:** Wakako Mori, Keisuke Yuzu, Nadine Lobsiger, Hideo Nishioka, Hisako Sato, Terumasa Nagase, Keiichi Iwaya, Mikael Lindgren, Tamotsu Zako

**Affiliations:** 1 Department of Chemistry and Biology, Graduate School of Science and Engineering, Ehime University, Ehime, 790-8577 Japan; 2 Institute for Chemical and Bioengineering, ETH Zürich, 8093 Zürich, Switzerland; 3 Application Management Department, JEOL Ltd, Tokyo, 196-8558 Japan; 4 Department of Metabolism and Endocrinology, Tokyo Medical University Ibaraki Medical Center, Ibaraki, 3000395 Japan; 5 Department of Pathology, SASAKI Institute, Kyoundo Hospital, Tokyo, 101-0062 Japan; 6 Department of Physics, Faculty of Natural Sciences, Norwegian University of Science and Technology, 7491 Trondheim, Norway

**Keywords:** Chemical biology, Protein folding, Proteins

## Abstract

Insulin balls, localized insulin amyloids formed at subcutaneous insulin-injection sites in patients with diabetes, cause poor glycemic control owing to impairments in insulin absorption. Our previous study has shown that some insulin balls are cytotoxic, but others are not, implying amyloid polymorphism. Interestingly, the patient with toxic insulin balls had been treated with antibiotic minocycline, suggesting a possible relationship between toxicity of insulin balls and minocycline. However, the direct effect of minocycline on the structure and cytotoxicity of the insulin amyloid is still unclear. Herein, we demonstrated that that minocycline at physiological concentrations induced degradation of insulin amyloids formed from human insulin and insulin drug preparations used for diabetes patients. Interestingly, the process involved the initial appearance of the toxic species, which subsequently changed into less-toxic species. It is also shown that the structure of the toxic species was similar to that of sonicated fragments of human insulin amyloids. Our study shed new light on the clarification of the revelation of insulin balls and the development of the insulin analogs for diabetes therapy.

## Introduction

Neurodegenerative disorders, such as Alzheimer’s disease and systemic amyloidosis, are associated with the accumulation of soluble and insoluble protein aggregates, which are central to their pathogenesis^1,2^. Amyloids typically show rigid, unbranched structures with diameters of 10 nm and lengths up to several micrometers, and possess a cross-β structure where the β-strands are aligned perpendicularly to the long axis^3^. Recent studies have also shown that oligomeric species are more toxic and cause diseases^4^.

Insulin is a 51-residue hormone that is important for the control of glucose metabolism and diabetes treatment^5^. Insulin is composed of two polypeptide chains, the A-chain (21 residues) and the B-chain (30 residues) linked together by two disulfide bonds^6,7^. It has previously been shown that insulin-derived amyloidosis or insulin balls could be formed in patients who inject insulins repeatedly at the same sites^8,9^ against the clinical guidelines^10^, which can cause poor glycemic control due to impaired insulin absorption^11–13^. A recent case report showed that some insulin balls are cytotoxic, whereas others are not, implying amyloid polymorphism^14^. Interestingly, the patient with toxic insulin balls had been administered with antibiotics including minocycline to treat diabetic gangrene with sepsis, suggesting a possible relationship between insulin balls and minocycline^14^. However, the direct effect of minocycline on the structure and cytotoxicity of insulin amyloids is still unclear.

In this study, we evaluated the structure and cytotoxicity of insulin amyloid incubated with minocycline as a model for insulin balls. Previously, we showed that bovine insulin could form two types of amyloids under different solvent conditions: toxic fibrils and less toxic filaments, which formed in the presence of a reducing reagent^15^. Certain amyloid-specific luminescent-conjugated oligothiophenes (LCOs) such as pFTAA, and benzothiadiazole (BTD) derivatives^16^, could discriminate between the two different insulin amyloids^17–19^. Herein, we also demonstrate that human insulin can form toxic amyloid from intact insulin ((i)-amyloid), and less toxic amyloid formed by insulin treated with reducing reagents ((r)-amyloid). Although (r)-amyloid was formed at unphysiological condition, our previous study showed that both types of amyloids were formed from some insulin preparations and also in patients’ insulin ball tissues^19^. Thus, we used these human insulin amyloids as a model of insulin balls. We also used amyloids formed from typical insulin analogs (lispro and detemir) as a model of insulin balls^19–21^. Doxycycline has been shown to have a degrading effect on the β2-microglobulin amyloid^22^. In this study, we showed that minocycline at physiological concentrations induced insulin amyloid degradation. The process involved the initial appearance of the toxic intermediate, which subsequently changed into harmless assemblies, implying a possible effect of cycline-related antibiotics on amyloidosis.

## Results and discussion

### Degradation of insulin amyloids by minocycline

**Figure 1:**
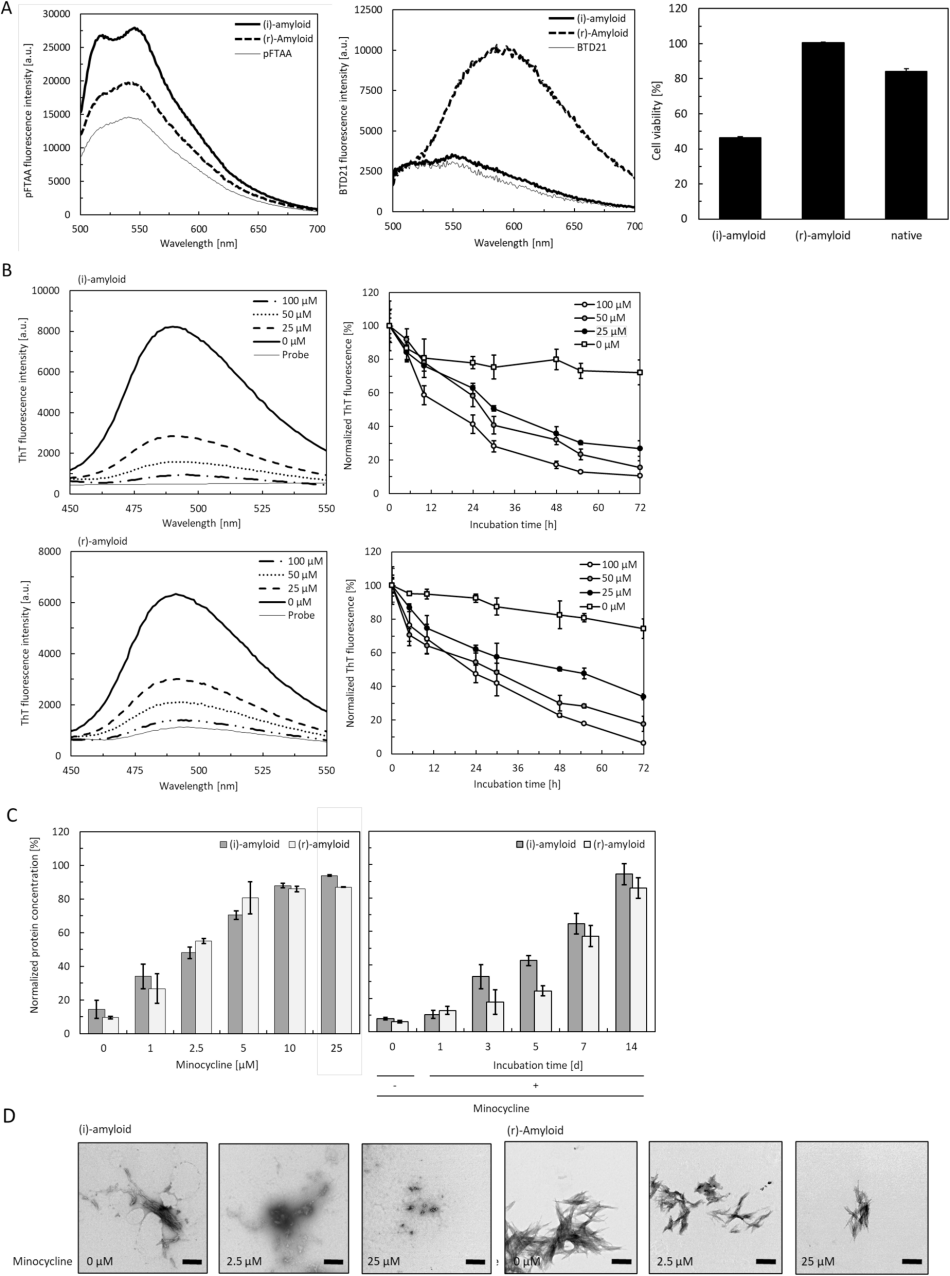
Degradation of two types of insulin amyloids by minocycline. (**A**) LCO assay and MTT assay of (i)-amyloid and (r)-amyloid. The left image is the spectrum of pFTAA, and the middle image is the spectrum of BTD21: (i)-amyloid (thick solid line), (r)-amyloid (thick dotted line), and probe (thin solid line). The right images show results of the MTT assay of (i)-amyloid, (r)-amyloid, and native insulin. All samples were quantified by BCA assay and were diluted to the same protein concentration (1 μM/well). The absorbance was normalized for PBS as 100%. (**B**) ThT assay of (i)-amyloid (upper) and (r)-amyloid (lower) incubated in the presence of 0–100 μM minocycline. Images on the left are the spectrum of ThT incubated with insulin amyloid for 72 h: amyloid only (thick solid line), 25, 50, and 100 μM minocycline (thick-dashed, thick dotted, solid-dashed lines, respectively), and probe (thin solid line). Pictures on the right are the plots of the intensities normalized for insulin amyloid as 100%: amyloid (white squares), and 25, 50, 100 μM minocycline (black, gray, and white circles, respectively). (**C**) BCA assay of supernatant samples to confirm the dependency of the degradation on minocycline concentration (upper) and incubation time (lower): (i)-amyloid (gray) and (r)-amyloid (white). Protein concentrations were normalized to 50 µM (all insulin amyloid concentrations). (**D**) TEM images of the structure of insulin amyloid after degradation. The three samples on the left area are (i)-amyloid, and the three samples on the right area are (r)-amyloid. The mature amyloid (left) was incubated with 2.5 (middle) or 25 µM (right) minocycline for 1 week. The scale bar is 200 nm.

To confirm the formation of two different types of amyloids from human insulin, the structure and toxicity of incubated insulin samples were evaluated using pFTAA and BTD21, and MTT assays, respectively. pFTAA and BTD21 can be utilized to recognize structural polymorphs of bovine insulin fibrils and filaments in fluorescence assays^19^. Following our previous approach, we carried out the formation of human insulin amyloids without TCEP ((i)-amyloid) or with a reducing reagent, TCEP ((r)-amyloid). As shown in Fig. 1A, the pFTAA fluorescence intensity of (i)-amyloid was higher than that of (r)-amyloid formed with the reducing reagent, TCEP. The BTD21 fluorescence intensity of (r)-amyloid was higher than that of (i)-amyloid. These results strongly support structural differences between the (i)-amyloid and (r)-amyloid forms. Consistent with our previous results on bovine insulin ^15^, the cytotoxicity of (i)-amyloid was high, whereas (r)-amyloid and native insulin were less toxic. These results indicate the formation of two types of amyloids from human insulin.

Next, we investigated the effect of minocycline on these two human insulin amyloids by comparing results of the thioflavin T (ThT) and BCA assays and by transmission electron microscopy (TEM) observation. As shown in Fig. 1B, the ThT fluorescence intensity of the samples incubated with minocycline showed an incubation time-dependent decrease, whereas those of the untreated (i)-amyloid and (r)-amyloid samples (incubated without minocycline) were higher. Furthermore, the ThT intensity decreased in a dose-dependent manner with minocycline concentration (Fig. S1). These results suggest that the two types of insulin amyloids were degraded by minocycline.

Degradation of insulin amyloids by minocycline was confirmed by measuring the amount of protein in the supernatant after centrifugation (Fig. 1C). The protein concentrations of the supernatants of minocycline-treated (i)-amyloid and (r)-amyloid increased in a dose-dependent manner (Fig. 1c, left) and in a time-dependent manner (Fig. 1C, right), suggesting that the insulin amyloids were degraded into smaller intermediates that appeared in the supernatant after centrifugation. TEM imaging was used to visualize the structure and appearance of the degraded products (Fig. 1D) and showed that the size of the degraded samples decreased. This result is corroborated with dynamic light scattering (DLS) measurements (Fig. S2). The size of degraded (i)-amyloid and (r)-amyloid decreased in a clear trend, depending on the minocycline concentration and incubation time. Interestingly, a significant difference in size between native insulin and the samples incubated with minocycline was observed at the highest concentration (25 µM) or for the longest time (14 days), suggesting that the final degradation products of the aggregates were larger than the native insulin.

### Cytotoxicity of minocycline-induced degradation products

**Figure 2:**
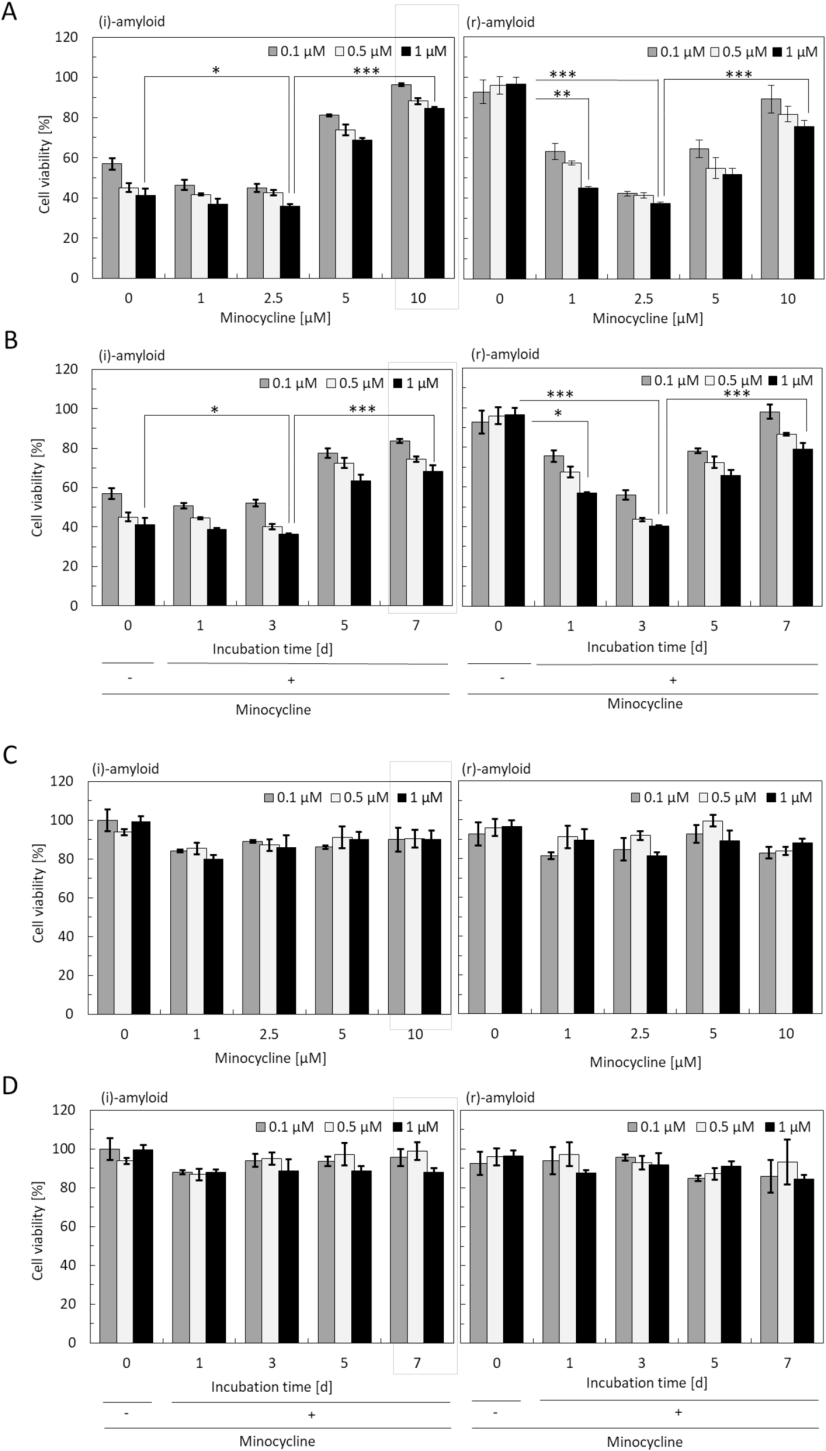
Cytotoxicity of the insulin amyloid samples in precipitate and supernatant using MTT assay against HeLa cells. Samples in the precipitate (**A**) and the supernatant (**C**) are (i)-amyloid (left) and (r)-amyloid (right) incubated with minocycline for 1 week at the indicated concentrations and centrifuged at 15,000 rpm for 15 min. Samples in the precipitate (**B**) and the supernatant (**D**) are (i)-amyloid (left) and (r)-amyloid (right) incubated with a fixed minocycline concentration (50 µM) for the indicated periods and centrifuged at 15,000 rpm for 15 min. Samples incubated without minocycline for 7 days were shown as controls [0 µM (**A**,**C**) and 0 day (**B**,**D**)]. All samples were quantified by BCA assay and were diluted to the same protein concentration: 0.1, 0.5, and 1 μM (white, gray, and black, respectively). The absorbance was normalized for PBS as 100%. (*P < 0.05, **P < 0.01, ***P < 0.005).

Toxic insulin balls, which are known to have developed in patients administered with minocycline, may contain degraded products, which in turn are more toxic than the insulin amyloid itself. To examine this hypothesis, the cytotoxicity of insulin amyloids incubated with minocycline on HeLa cells was examined using the MTT assay. For this, we used centrifugation-separated supernatant and precipitated samples, in which the protein concentrations were adjusted. The cytotoxicity of minocycline was confirmed to be negligible in the concentration range 0.5–2 μM (Fig. S3). Figure 2A shows the cytotoxicity of precipitate samples formed by insulin amyloids incubated with different concentrations of minocycline. Interestingly, the highest cytotoxicity of degraded products of both types of amyloid was observed after incubation with 2.5 µM minocycline, although the less toxic (r)-amyloid became more toxic upon incubation with minocycline. Unexpectedly, the (r)-amyloid incubated at higher minocycline concentrations showed significantly decreased cytotoxicity. To confirm this observation, we also analyzed amyloids incubated with minocycline for 1, 3, 5, and 7 days to check whether a longer incubation period of insulin amyloids with minocycline potentially decreases cytotoxicity. The cytotoxicity of (i)-amyloid was maximal after 3 days of incubation and then progressively decreased to eventually disappear after 7 days (Fig. 2B, left panel). More interestingly, the degraded (r)-amyloid formed toxic products after a shorter incubation time (3 days), similar to the result of (i)-amyloid. Similar results were obtained for PC12 cells (Fig. S4A), suggesting that the increased cytotoxicity of degradation products is not cell-specific. These results indicated that both types of amyloid, and the less toxic (r)-amyloids, in particular, could become toxic upon degradation by minocycline. Furthermore, we examined the cytotoxicity of the supernatant after centrifugation. Interestingly, the supernatant samples were not toxic to either HeLa cells or PC12 cells (Fig. 2C,D and Fig. S4B), whereas uncentrifuged samples showed similar toxicity to the precipitated samples (Fig. S5). These results also indicated that the primal toxic intermediates existed in the precipitate, and that the soluble species in the supernatant after centrifugation were not toxic.

### The structural difference between the toxic and less-toxic products

**Figure 3:**
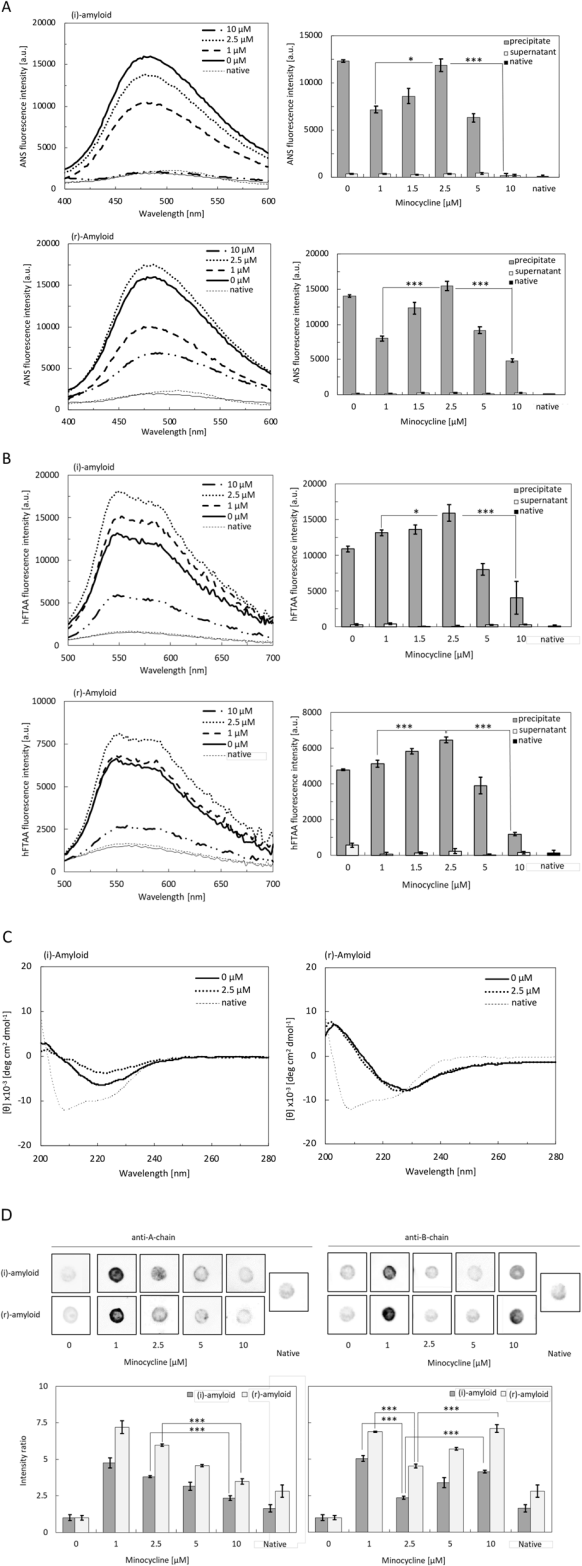
Structural analysis of insulin amyloids incubated with minocycline. (i)-amyloid (upper) and (r)-amyloid (lower) were incubated with minocycline for 1 week at the indicated concentrations, and the precipitate and supernatant samples were stained by ANS (**A**) and hFTAA (**B**). Graphs on the left are ANS spectrum with: amyloid only(thick solid line), 1, 2.5, and 10 μM minocycline (thick dashed, thick dotted, and solid-dashed lines respectively), ANS probe only (thin solid line), and native insulin (thin dotted line). Graphs on the right are the fluorescence values in emission wavelength (ANS: 480 nm; hFTAA: 560 nm): precipitate (gray), supernatant (white), and native insulin (black). Protein concentrations were adjusted to 5 µM. (**C**) Far-UV CD spectra of the precipitate samples of (i)-amyloid (left) and (r)-amyloid (right): amyloid (thick solid line), 2.5 μM minocycline (thick-dotted line), and native insulin (thin dotted line). (**D**) Dot blot assay of the degraded samples in precipitate by anti-insulin A chain and B chain antibody as a primary antibody. (i)-amyloid and (r)-amyloid were incubated with minocycline for 1 week at the indicated concentrations; 5 μM precipitate samples after centrifugation were used. Graphs show the analysis of luminescence intensities by anti-A-chain antibody (left) and anti-B-chain antibody (right) by ImageJ: (i)-amyloid (gray) and (r)-amyloid (white). The intensities were normalized for each mature amyloid as 100% (*P < 0.05, **P < 0.01, ***P < 0.005).

The structural differences between toxic and less-toxic degraded products were investigated using 1-anilinonaphthalene 8-sulfonate (ANS) and hFTAA assays, circular dichroism (CD) spectroscopy, and dot blots with anti-insulin antibodies. Notably, the same protein concentration (5 µM) was used in all these assays. First, we investigated the change in the exposed hydrophobic residues of the degraded precipitates using the hydrophobicity probe, ANS (Fig. 3A)^23–25^. The ANS fluorescence of (i)-amyloid treated with 2.5 μM minocycline was higher than that of (i)-amyloid treated with a higher minocycline concentration. No increase in the ANS fluorescence intensity was observed for the supernatant samples. Similar results were obtained for the insulin samples treated with minocycline for different incubation times (Fig. S6A). These results imply that the toxic species have exposed hydrophobic regions, and that the surface hydrophobicity might be related to the toxicity of degraded products. Notably, the ANS fluorescence intensities of the amyloids decreased after the addition of 1 μM minocycline, possibly due to the initial degradation reactions.

We also investigated the structure of the precipitate using the LCO hFTAA, which is a fluorescence amyloid ligand known to detect both pre-fibrillar and fibrillar structures^26,27^ (Fig. 3B). The hFTAA intensity of the toxic species formed in the presence of 2.5 μM minocycline was higher than that of less toxic intermediates formed at both lower and higher concentrations of minocycline, whereas the hFTAA intensity of the supernatant samples did not increase. Similar results were obtained for the insulin samples treated for different incubation times (Fig. S6B). These results suggest that the toxic species formed from both (i)-amyloid and (r)-amyloid by minocycline might have the local fibrillar structure.

Next, we examined the secondary structures of the untreated amyloids and the toxic species (Fig. 3C). The CD spectra revealed that there was no significant difference between untreated amyloids and the toxic species formed in the presence of 2.5 μM minocycline, indicating that the β-sheet structure of toxic species remained even after degradation. This result is consistent with the result obtained by hFTAA showing the presence of fibrillar/pre-fibrillar structure of the toxic species (Fig. 3B). This result is also consistent with a previous study reporting that there was no significant difference in the secondary structure between the fragments formed by the sonicated α-synuclein aggregates and mature aggregates^28^.

The surface structure was then examined by dot blot assay using anti-insulin A and B chain antibodies (Fig. 3D). Interestingly, the effect of minocycline concentration was different between the anti-A chain and the anti-B chain. Recognition by the anti-A chain became weaker when more minocycline was added. In contrast, recognition by the anti-B chain was the weakest for the toxic species formed by 2.5 μM minocycline. Similar results were obtained for the precipitated insulin samples incubated with minocycline for various incubation times (Fig. S6C). The dot intensities of the precipitate were increased by the addition of 1.0 μM minocycline, possibly due to the increase of the epitope by the initial degradation. The insulin fibrils have been suggested to contain the inner structure formed by the B-chain^29^. Thus, it is plausible that the B-chain motifs were initially exposed by degradation with minocycline, whereas the A-chain variants were continuously degraded. This may be partially supported by studies showing that antibody recognition differs between mature amyloids and oligomers^30,31^.

**Figure 4:**
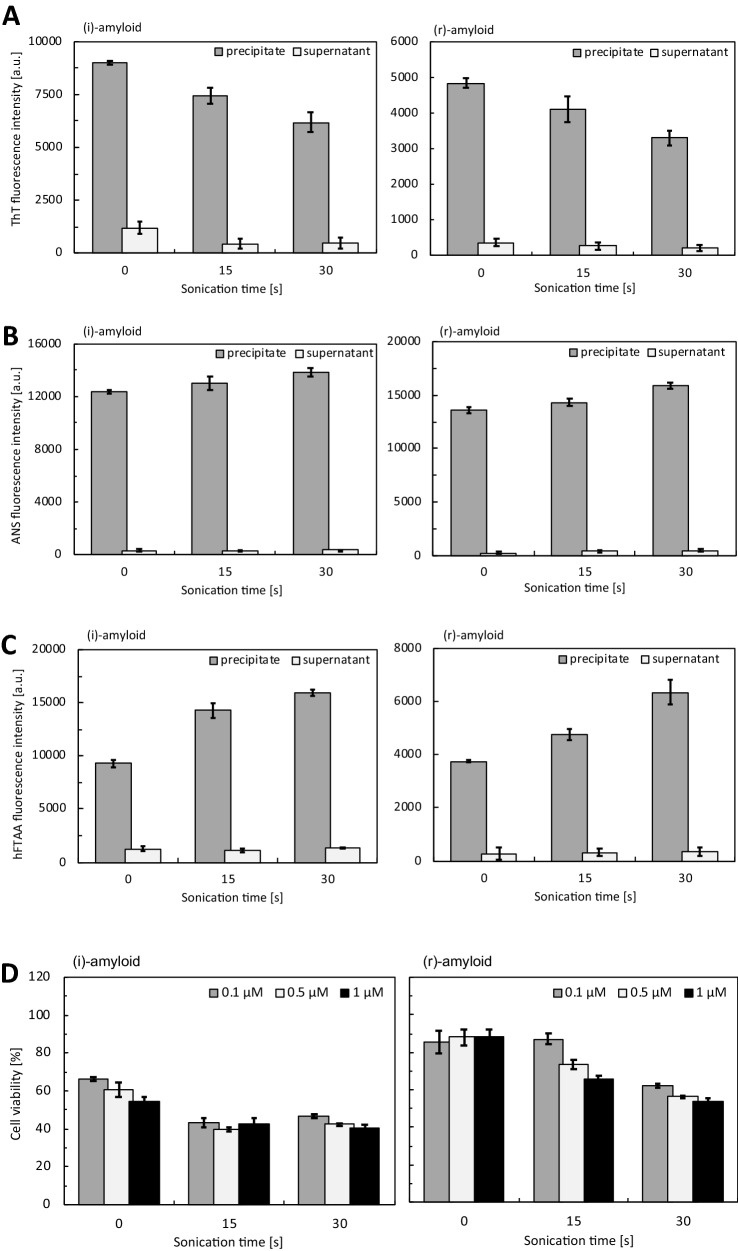
Structure and toxicity of the sonicated insulin amyloids. The (i)-amyloid (left) and (r)-amyloid (right) samples were sonicated for the indicated time, and the precipitate and supernatant samples were evaluated by ThT (**A**), ANS (**B**), and hFTAA (**C**). Peak fluorescence values at 490 nm (ThT), 480 nm (ANS), and 560 nm (hFTAA) are shown; precipitate (gray), and supernatant (white). Protein concentrations were adjusted to 5 µM. (**D**) Cytotoxicity of the sonicated (i)-amyloid (left) and (r)-amyloid (right). All samples were quantified by BCA assay and were diluted to the same protein concentration: 0.1, 0.5, and 1 μM (white, gray, and black, respectively) The absorbance was normalized for PBS as 100%.

Previous studies have shown that protein amyloids (e.g., tau, α-synuclein) could be physically fragmented by sonication, and that the cytotoxicity of the fragments was greater than that of mature amyloid^28,32,33^. The fragmentation of insulin fibrils by sonication has also been reported^34^. Based on these findings, we hypothesized that a temporary increase in cytotoxicity could be due to the degradation of insulin amyloids by minocycline into toxic species. To further support this idea, we investigated the structure and toxicity of the fragments of (i)-amyloid and (r)-amyloid formed by sonication as a model of toxic species caused by degradation with minocycline. A decrease in ThT fluorescence intensity was observed for the sonicated samples of both types of insulin amyloids (Fig. 4A). Moreover, the fluorescence intensities of ANS and hFTAA increased upon sonication, indicating expose of hydrophobic residues and fibrillar structures (Fig. 4B,C). These results are consistent with those of the samples by minocycline (Fig. 3), supporting the idea that insulin amyloids degraded by minocycline share common structural properties with the sonicated fragments. These results are also consistent with studies showing that both oligomers and pre-fibrils of Aβ42 could be detected with hFTAA fluorescence^27,35^ and that sonicated α-synuclein aggregates exhibited higher ANS fluorescence intensity than mature amyloid^28^. Notably, the fluorescence intensity of the samples in the supernatant after centrifugation did not increase; importantly, the cytotoxicity of sonicated insulin amyloids for HeLa cells did increase in a sonication time-dependent manner (Fig. 4D and Fig. S7 for PC12 cells). These results support that the formation of toxic species by minocycline is similar to those formed by sonication.

Taken together, the toxic species formed by degradation by minocycline exhibited more hydrophobic residues and a local fibrillar structure on the surface than the less-toxic species, and shared similar properties with the samples formed by sonication.

### Identification of the binding site of minocycline in insulin amyloids

**Figure 5:**
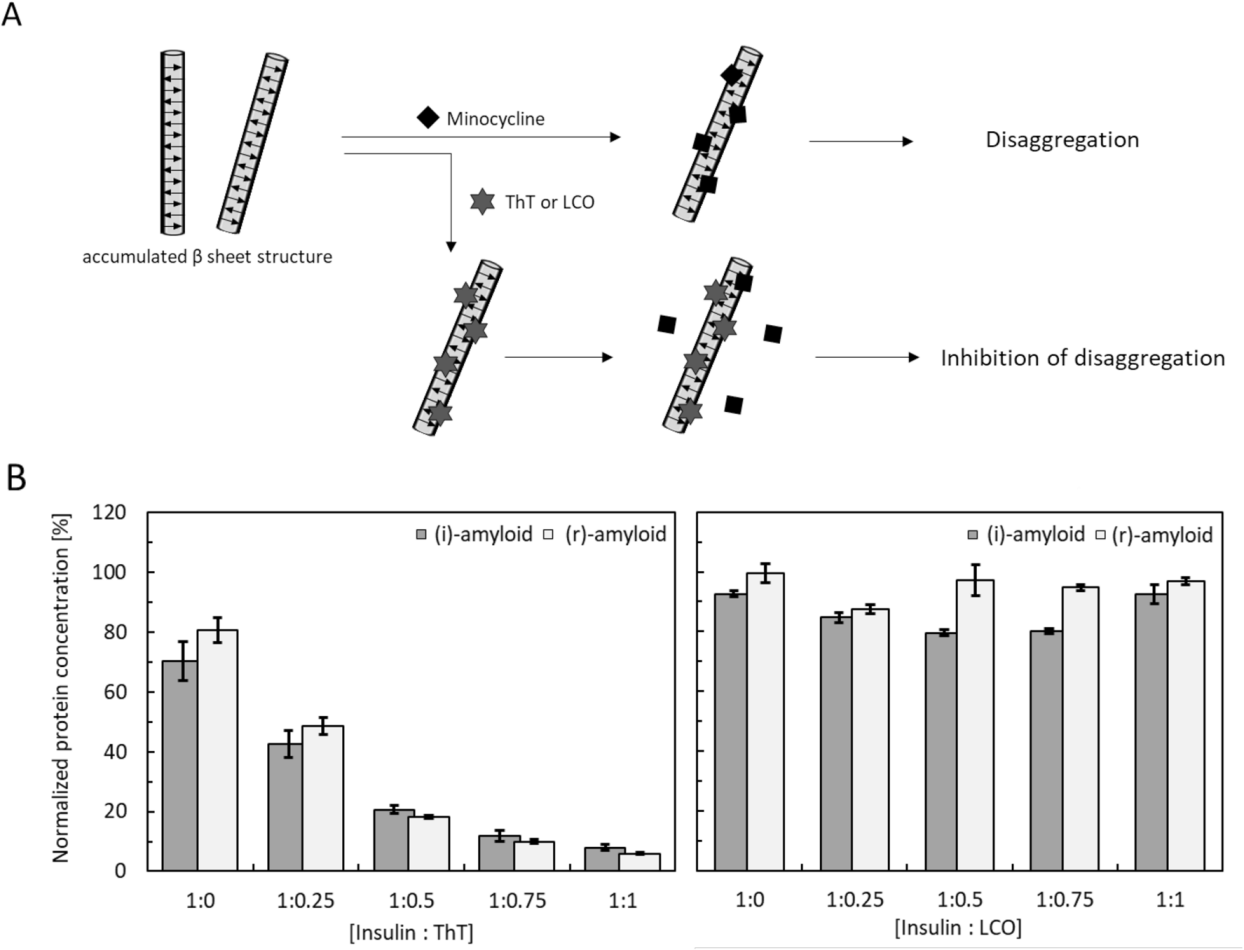
Identification of the binding site of minocycline to insulin amyloids. (**A**) Scheme of a model of insulin amyloid blocked by fluorescence probes at the minocycline binding site. (**B**) BCA assay of supernatant samples of (i)-amyloid and (r)-amyloid blocked by ThT (left) and LCOs (right) to confirm the identification of the binding site of minocycline in insulin amyloid: (i)-amyloid (gray) and (r)-amyloid (white). pFTAA and BTD 21 were used for (i)-amyloid and (r)-amyloid, respectively. Protein concentration was normalized for 50 µM (all insulin amyloid concentration).

The binding sites of antibiotics on insulin amyloids remain unclear. The stacked β-sheet structure of insulin amyloids contains hydrophobic residues^36^, and minocycline could bind to hydrophobic sites^37^. Thus, a possible hypothesis is that the binding site of minocycline is the stacked β-sheet structure. To test this, we investigated insulin amyloids and used fluorescence amyloid ligands, such as ThT, pFTAA, and BTD21 to block the stacked β-sheet structure followed by attempted incubation with minocycline^38^, with the assessment of the degradation via BCA assay (Fig. 5A). We expected that the protein concentration in the supernatant would remain low if these probes inhibited the binding of minocycline to insulin amyloids. Accordingly, degradation was inhibited at higher ThT concentrations, indicating that the binding site of ThT and minocycline is similar (Fig. 5B). However, interestingly, the amyloid samples were degraded by minocycline in the presence of pFTAA (for (i)-amyloid) and BTD21 (for (r)-amyloid), suggesting that the binding site of minocycline and the recognition sites of pFTAA/ BTD21 are different. It should be pointed out that the effect of minocycline on the binding of ThT to insulin amyloids was negligible, because the ThT fluorescence values did not change with the addition of minocycline (Fig. S8). Thus, binding of ThT to insulin amyloids may be stronger than that of minocycline. These results indicate that ThT inhibits the binding of minocycline to insulin amyloids and that the binding site of minocycline in insulin amyloids is the accumulated β-sheet structure.

### Formation of amyloid formed by insulin preparations and degradation by minocycline

**Figure 6:**
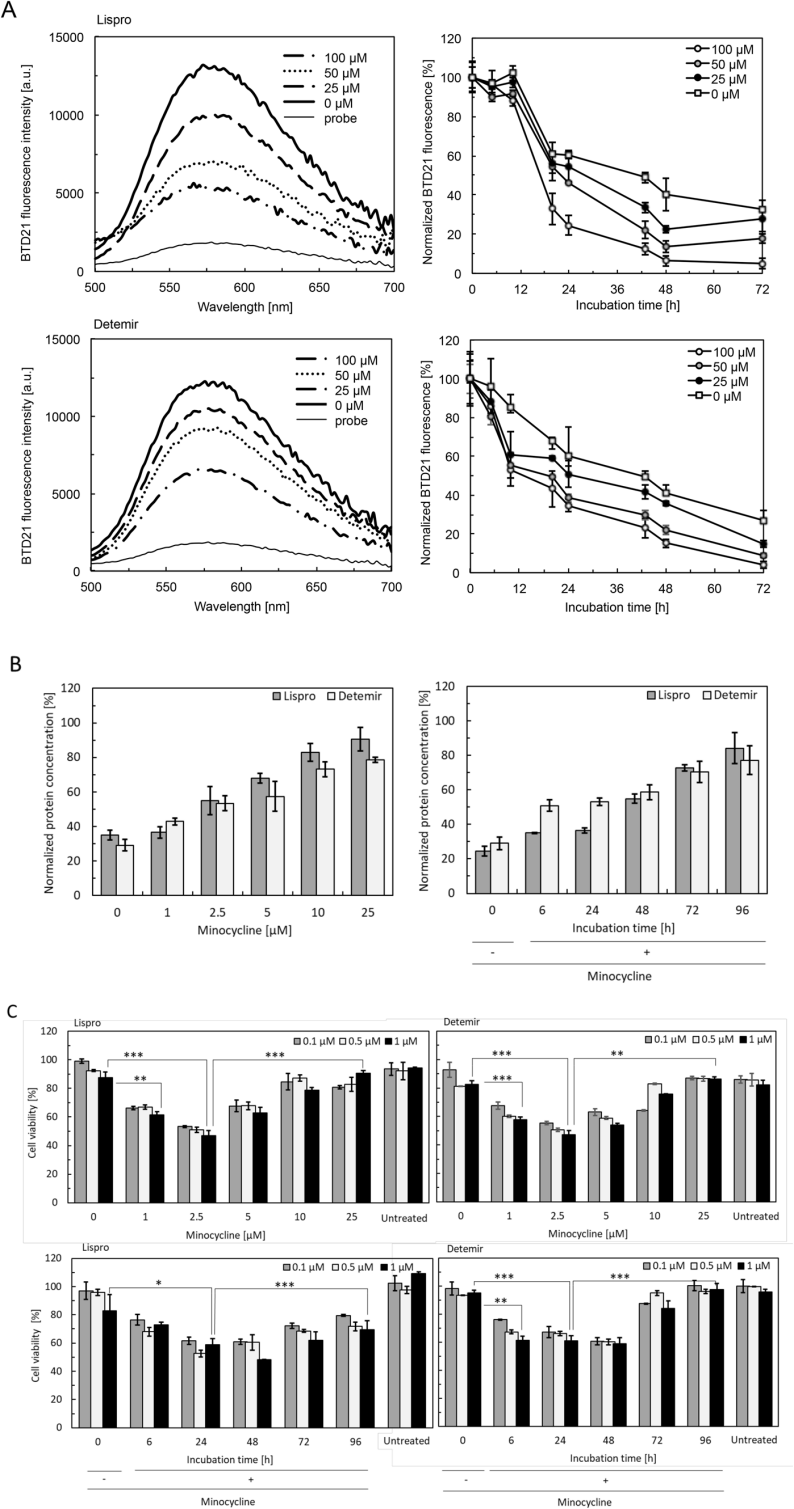
Degradation of insulin amyloids formed by insulin preparations (lispro and detemir) by minocycline. (**A**) BTD21 assay of lispro (upper panel) and detemir (lower panel) incubated in the presence of 0–100 μM minocycline. The left figures are the spectrum of BTD21 of insulin amyloid incubated for 72 h: amyloid (thick solid line), 25, 50, and 100 μM minocycline (thick-dashed, thick dotted line, solid-dashed lines, respectively), and BTD21 probe (thin-solid line). The right figures are the plots of the intensities normalized for insulin amyloid as 100%: amyloid (white squares) and 25, 50, and 100 μM minocycline (black, gray, white circles, respectively). (**B**) BCA assay of supernatant samples to confirm the dependency of the degradation on minocycline concentration (upper) and incubation time (lower): lispro (gray) and detemir (white). Protein concentrations were normalized for 50 µM (all insulin amyloid concentrations). (**C**) Cytotoxicity of degraded samples using MTT assay against HeLa cells. Lispro (upper left) amyloid and detemir (upper right) were incubated with minocycline for 1 week at the indicated concentrations. Lispro (lower left) and detemir (lower right) were incubated with a fixed minocycline concentration (50 µM) for the indicated periods. 0.1, 0.5, and 1 μM are indicated by gray, white, and black, respectively. The absorbance was normalized for PBS as 100%. (*P < 0.05, **P < 0.01, ***P < 0.005).

Many diabetic patients are administered insulin preparations for the control of blood glucose levels. Various insulin analogs with improved activity have been used^39–41^. In this study, the minocycline-induced degradation of amyloid formed by insulin preparations was examined by BTD21, BCA, and MTT assays as described above. Since our previous studies showed that the insulin analogs lispro and detemir formed less-toxic amyloids that were detected with BTD21^19^, these analogs were used for further study. The amyloids formed by lispro and detemir were incubated with minocycline and were evaluated based on their fluorescent signal in the BTD21 assay (Fig. 6A). As shown in the figure, the BTD21 fluorescence of the lispro and detemir samples incubated with minocycline exhibited an incubation time-dependent decrease, whereas the samples without minocycline showed relatively high fluorescence. The decrease in fluorescence was more significant at higher minocycline concentrations. These results indicate that minocycline has a degrading effect on amyloids formed by insulin analogs at room temperature.

To further confirm the minocycline-induced degradation of amyloids induced by insulin analogs, the protein concentration in the supernatant after centrifugation was quantified. There was a dose-dependent increase in protein concentration in the supernatant with minocycline, indicating degradation by minocycline (Fig. 6B, left). This was also supported by degradation over the extended incubation time (Fig. 6B, right). Samples that were incubated with 25 µM minocycline and for 96 h contained nearly 90% of the protein in the supernatant, indicating high degradation yields by minocycline. Importantly, insulin amyloids formed by analogs were degraded at a lower concentration of minocycline and a shorter incubation time than human insulin amyloids. In addition to these analogs, glargine 1, glargine 2, and glulisine were incubated with minocycline and were evaluated based on their fluorescent signal in the pFTAA and BTD21 assay (Fig. S9). The amyloids formed from these insulin analogs also showed decreased fluorescence upon incubation with minocycline. Incubation of these three analogs with minocycline also led to an increased protein concentration in the supernatant, depending on the concentration and incubation time with minocycline (Fig. S10). A decrease in the ThT intensity of the five insulin analogs (lispro, detemir, glargine 1, glargine 2, and glulisine) by minocycline was also observed (Fig. S11). These results strongly suggest that insulin amyloids formed by various analogs could be degraded by minocycline.

Evaluation of the cytotoxicity of degraded insulin analog amyloids is important to elucidate the mechanism of the toxicity of insulin balls. Cytotoxicity was therefore quantified in MTT assay with HeLa cells and the effects of minocycline concentration and incubation time were tested. The highest cytotoxicity of lispro and detemir degradation products was observed after incubation with 2.5 µM minocycline (Fig. 6C, upper panel), although the toxicity decreased at higher minocycline concentrations. The cytotoxicity of lispro and detemir was maximal after approx. 48 h of incubation and then progressively declined until 96 h (Fig. 6C, lower panels). These results are consistent with those obtained for human insulin amyloids (Fig. 2). We also showed that the degraded products formed by lispro and detemir were cytotoxic against PC12 cells, suggesting that the toxicity is not cell-specific (Figure. S12A). The cytotoxicity of amyloids formed by glargine 1, glargine 2, and glulisine initially increased, and then decreased against both HeLa and PC12 cells as the degradation reactions by minocycline proceeded (Fig. S12B). These results indicate that degradation of insulin preparations initially produces toxic species and then forms low-toxicity products similar to the case of human insulin.

## Conclusion

**Figure 7:**
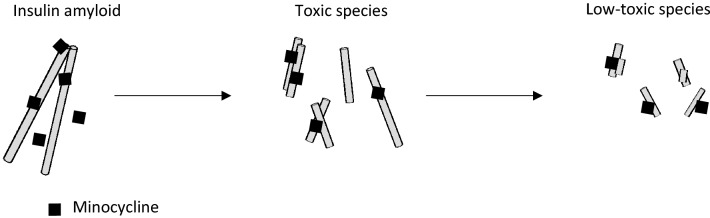
Schematic illustration for the degradation of insulin amyloids by minocycline.

In this study, we demonstrated the degradation of human insulin amyloids by minocycline (Fig. 7). Interestingly, increased cytotoxicity towards two different cells, HeLa and PC12, was observed for the intermediates of the degraded human insulin amyloids and insulin analogs, with low-toxicity products produced as the end products. Responses to hFTAA, ANS, and anti-insulin antibodies suggested structural differences between toxic and low-toxic species. The toxic species are present in the precipitation, suggesting that relatively large and insoluble insulin amyloid intermediates were cytotoxic. According to the PK profile of the manufacturer’s instruction, the serum concentration of minocycline after 200 mg intravenous injection is 4.4 μg/mL (8.9 μM) with a half-life of 6 h. We have also confirmed that degradation of insulin amyloids could also be observed in the presence of low concentrations of minocycline ranging from 1 to 10 μM (Fig. S13). It is also noted that degradation of insulin amyloids into toxic species was observed at this concentration range. Thus these degradation reactions are expected to occur in vivo because the experimental conditions used in this study are similar to the biological conditions in terms of the administered minocycline concentration, pH, and temperature. Although future research should be concerned with a more detailed comparison of the structures of toxic species and low-toxic end products to clarify the mechanisms involved in the toxicity of insulin balls, insights into the relationship between insulin amyloids and minocycline may shed new light on the clarification of the revelation of insulin balls and the development of insulin analogs for diabetes therapy.

## Methods

### Materials

Human insulin was purchased from Wako (Fujifilm, Tokyo, Japan). Tris (2-carboxyethyl) phosphine hydrochloride (TCEP) was obtained from Sigma-Aldrich (St. Louis, MO). The following insulin analogs were used: insulin lispro (HUMALOG, Eli Lilly Japan, Kobe, Japan), insulin detemir (LEVEMIR, Novo Nordisk Pharma, Tokyo, Japan), insulin glargine (GLARGINE [glargine 1], Eli Lilly Japan, and LANTUS [glargine 2], Sanofi, Tokyo, Japan), insulin glulisine (APIDRA, Sanofi). All preparations were supplied as solutions. Minocycline was obtained from Pfizer Inc. pFTAA, hFTAA, and BTD21 prepared as described previously^39,40^. All other reagents were of analytical grade. Aqueous solutions were prepared with deionized Milli-Q water (Millipore, Billerica, MA, USA).

### Preparation of insulin amyloids

All protein samples were prepared immediately before further experiments were performed. Insulin was dissolved at a concentration of 20 mg/mL in 100 mM HCl, and then 100 mM NaOH was added for neutralization. The solution was immediately diluted in PBS at a final protein concentration of 2 mg/mL (344 µM). The formation of (i)-amyloid was induced by incubating the insulin solution at 37 °C for 96 h with agitation at 600 rpm^44–46^. The formation of (r)-amyloid was induced by incubating the insulin solution with 50 mM TCEP at 37 °C for 96 h with agitation at 600 rpm as described with a slight modification^15^. Samples of 2 mg/mL incubated insulin were dialyzed against PBS using a Slide-A-Lyzer Mini dialysis units (10,000 MWCO; Pierce) to remove native insulin and TCEP. The (i)-amyloid and (r)-amyloid concentrations were determined by BCA assay (Pierce™ BCA Protein Assay Kit) after dialysis.

The insulin analog amyloids were formed as described with a slight modification^19^. Each insulin preparation solution was diluted with PBS into 344 µM without purification, according to the concentrations shown in the manufacturers’ instructions. Then the solutions were incubated at 60 °C for 48 h without agitation.

The 2 mg/mL (i)-amyloid and (r)-amyloid were sonicated using an ultrasonic horn. Samples were subjected to 5 s bursts on ice with 5 s intervals. The sonication intensity was maintained at 50% to avoid foaming of the sample. After the sixth burst, the samples were incubated on ice for 30 min before being used for experiments.

### The reaction of insulin amyloids with minocycline

Degradation products were prepared to investigate the effect of incubation time and concentration dependency. To assess the effect of incubation time, 50 μM insulin amyloids were incubated with 25 μM minocycline in PBS at 37 °C (pH 7.4). Degradation dependence on the minocycline concentration was assessed by incubating the insulin amyloids (50 μM) with minocycline (different concentrations) in PBS at room temperature (pH 7.4). To prepare the precipitated and supernatant samples, the degraded products produced after incubation were centrifuged at 15,000 rpm for 15 min. Protein concentrations in the supernatant were analyzed in a BCA assay (Pierce™ BCA Protein Assay Kit). Precipitated and supernatant samples were diluted with PBS to equal the protein concentration of each sample.

### Fluorescence measurement

Fluorescence spectra were measured at 25 °C with a microplate reader (BioTek, VT, US) after combining 16 µL of 50 μM incubated insulin amyloids mixture with 144 µL of a solution containing 1.1 µM ThT, LCO, BTD21, or ANS in PBS. Each sample was prepared at 5 μM protein and 1 μM probe concentrations. The excitation wavelength of ThT was set to 420 nm and the emission was measured at 490 nm. The excitation wavelength of pFTAA and BTD21 was set to 470 nm, and the emissions of pFTAA and BTD21 were measured at 540 and 580 nm, respectively. The excitation wavelength of ANS was set to 360 nm, and the emission was measured at 480 nm.

### TEM

TEM measurements were performed using a JEM-1400 Flash transmission electron microscope (JEOL, Tokyo, Japan) operated at 120 kV. Samples were diluted with distilled water and negatively stained with 2% (w/v) uranyl acetate solution on copper grids (400 150-mesh) covered by carbon-coated Formvar film (JEOL DATUM, Tokyo, Japan).

### MTT assay

Cell viability was determined using an MTT cell proliferation kit (Roche, Basel, Switzerland) as described^15,19^. HeLa cells were maintained in EMEM medium with 10% fetal bovine serum, penicillin (100 U/mL), and streptomycin (100 mg/mL) in 5% CO_2_ at 37 °C. Cells were plated in 96-well plates at a density of 25,000 cells/well and grown overnight. Cells were subsequently incubated in 100 µL medium in the absence (control) and presence of insulin amyloids and degraded products at different concentrations. After 24 h of incubation, 10 µL of the MTT reagent was added to each well. Cells were incubated for 4 h at 37 °C. The reaction was stopped by adding 100 µL of 10% SDS in 10 mM HCl. Plates were read with a microplate reader (BioTek, VT, USA) at 562 nm. Each data point is an average of triplicates.

### Far-ultraviolet CD

The far-ultraviolet (UV) CD spectra of insulin samples 0.15 mg/mL were recorded using a Jasco-720 spectropolarimeter (Jasco, Tokyo, Japan) at 25 °C with a thermostated-controlled cell holder and a 1.0 mm path-length quartz cuvette. The CD spectrum of distilled water was subtracted from the sample spectra for background correction. The spectra were expressed as mean residue molar ellipticity [θ].

### Dot blot

Insulin sample (2 μL) was spotted onto a nitrocellulose membrane (0.22 μm, GE Healthcare Life Sciences, Buckinghamshire, UK). The membrane was blocked with 5% skim milk in Tris-buffered saline (TBS) containing 0.01% Tween 20 for 1 h at room temperature. Subsequently, the membrane was incubated with primary insulin antibody (1:2000, Santa Cruz Biotechnology, Texas, US) for 24 h and incubated with the secondary anti-goat IgG antibody (1:10,000, R&D Systems, Minneapolis, MN, USA) for 1 h. Proteins were visualized using the ECL blotting detection kit (Bio-Rad, Hercules, CA, USA) according to the manufacturer’s instructions. Luminescence was detected with a Las 4000 mini luminescent Image Analyzer (Fujifilm, Tokyo, Japan) using the Image Reader Las 4000 software. ImageJ was used to determine the intensity of each dot, which was used to quantify the amount of antibody on the membrane.

### Blocking the binding sites of minocycline to human insulin amyloids

Human insulin amyloids (100 μM) were incubated with ThT, pFTAA, or BTD21 (0, 25, 50, 75, 100 μM) in PBS at 37 °C (pH 7.4). After 24 h of incubation, 25 μM minocycline was added to each sample. These samples were incubated for 1 week at 37 °C. Solutions were centrifuged at 15,000 rpm for 15 min. Protein concentrations in the supernatant were analyzed using a BCA assay (Pierce™ BCA Protein Assay Kit).

## Supplementary Information

Supplementary Information

## Data Availability

All data generated or analyzed during this study are included in this published article (and its Supplementary Information files). This study contains supporting information Figure S1–S13. Correspondence and requests for materials should be addressed to T.Z.
