# Supplementary material for: Degradation of insulin amyloid by antibiotic minocycline and formation of toxic intermediates
Source: Sci Rep. 2021 Mar 25;11:6857. doi: 10.1038/s41598-021-86001-y (PMC7994847; doi:10.1038/s41598-021-86001-y)
Supplement: Supplementary file 1 — Supplementary Information [file 41598_2021_86001_MOESM1_ESM.pdf]

Electronic Supplementary Information

Degradation of insulin amyloid by antibiotic minocycline and formation of toxic intermediates

Wakako Mori[1], Keisuke Yuzu[1], Nadine Lobsiger[1,2], Hideo Nishioka[3], Hisako Sato[1], Terumasa Nagase[4], Keiichi Iwaya[5], Mikael Lindgren[6] and Tamotsu Zako[1*]

[1] Department of Chemistry and Biology, Graduate School of Science and Engineering, Ehime University, Ehime, Japan.

[2] Institute for Chemical and Bioengineering, ETH Zürich, Wolfgang-Pauli-Strasse 10, CH-8093 Zürich, Switzerland

[3] Application Management Department, JEOL Ltd.

[4] Department of Metabolism and Endocrinology, Tokyo Medical University Ibaraki Medical Center, Ibaraki, Japan

[5] Department of Pathology, SASAKI Institute, Kyoundo Hospital, Tokyo, Japan

[6] Department of Physics, Faculty of Natural Sciences, Norwegian University of Science and Technology, Trondheim, Norway.

*Corresponding author: Tamotsu Zako

E-mail: zako.tamotsu.us@ehime-u.ac.jp

Running title: Degradation of insulin amyloid by antibiotic minocycline

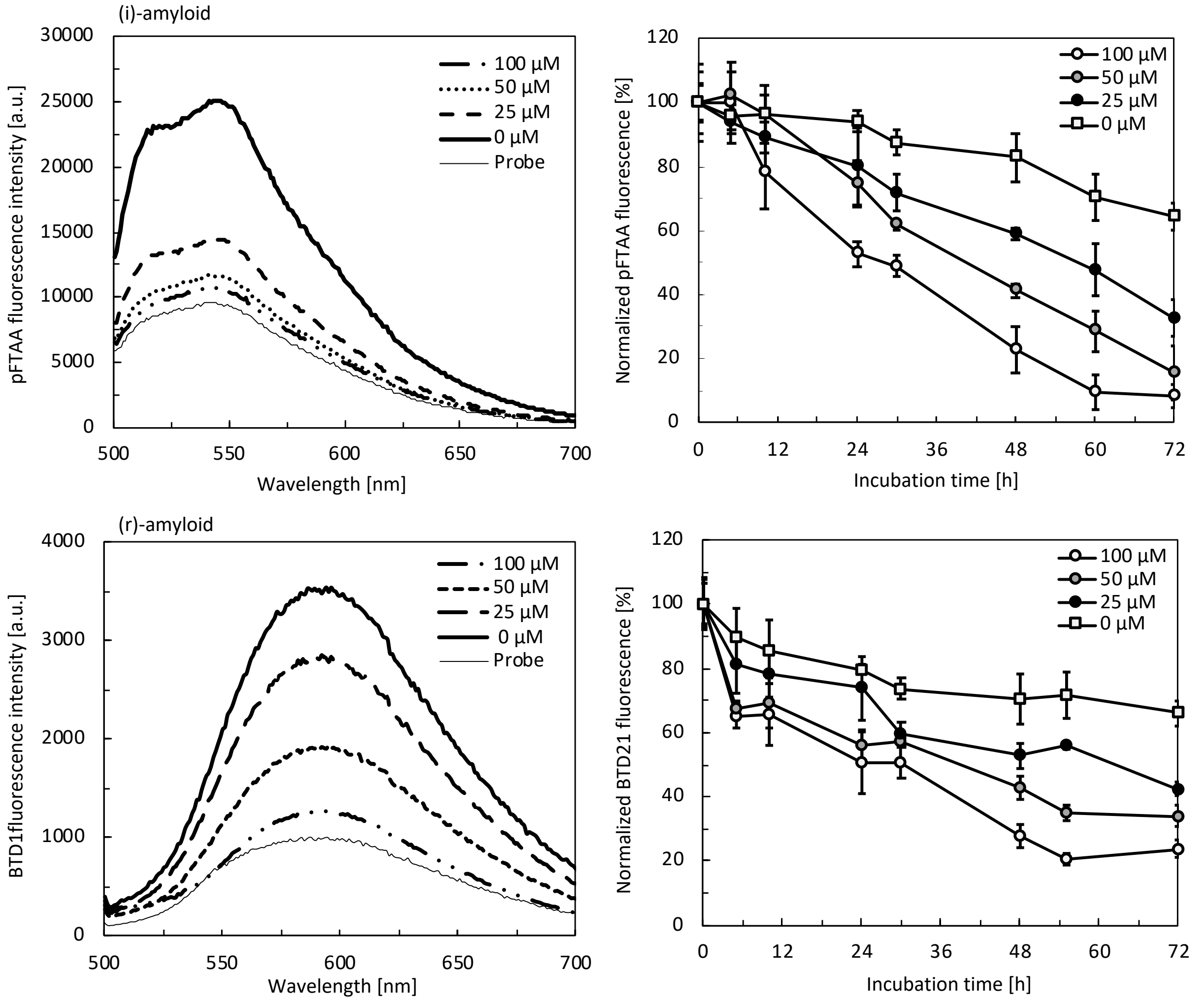

Figure S1 pFTAA and BTD21 assay for the insulin amyloid samples incubated with minocycline. pFTAA and BTD21 assay of (i)-amyloid (upper) and (r)-amyloid (lower) incubated in the presence of 0-100 µM minocycline. The left images are the spectrum of pFTAA or BTD21 of insulin amyloids incubated for 72 h: probe (thin solid line), amyloid (thick solid line), and 25, 50, and 100 µM minocycline (thick-dashed, thick-dotted, and solid-dashed line, respectively). The right pictures are plots of the intensities normalized for insulin amyloid as 100%: amyloid (white squares) and 25, 50, and 100 µM minocycline (black, gray and white circles, respectively).

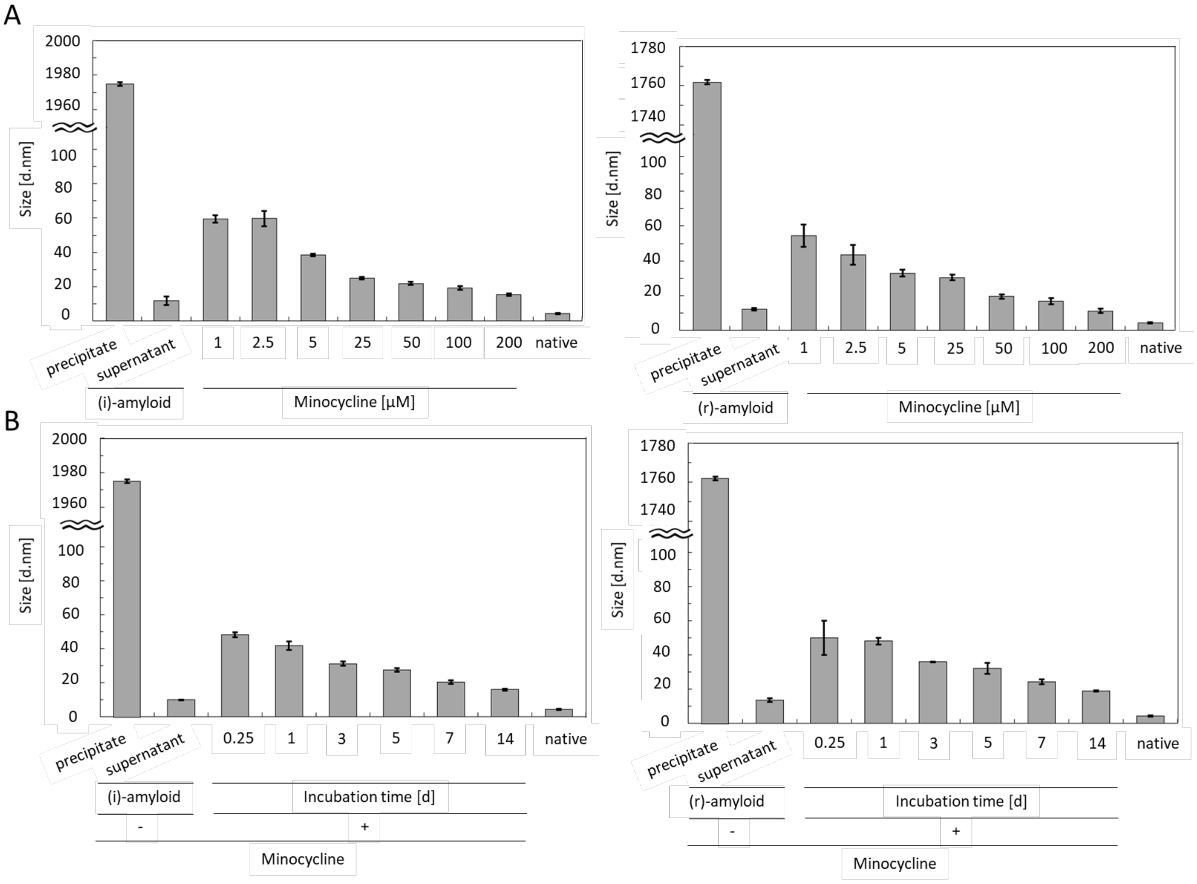

Figure S2 Dynamic light Scattering (DLS) measurement for the insulin amyloid samples incubated with minocycline.

(A) (i)-amyloid (upper left) and (r)-amyloid (upper right) were incubated with minocycline for 1 week at the indicated concentrations. The solutions were centrifuged at 15,000 rpm for 15 min. The protein concentration in the supernatant was adjusted to the same protein concentration (17.2 μM (0.1 mg/mL)) as determined by a BCA assay. The size was analyzed using Zetasizer Nano-ZS (Malvern, Worcestershire, UK). The average values of three repeated measurements are shown. **(B)** (i)-amyloid (upper left) and (r)-amyloid (upper right) incubated with a fixed minocycline concentration (50 μM) for the indicated periods.

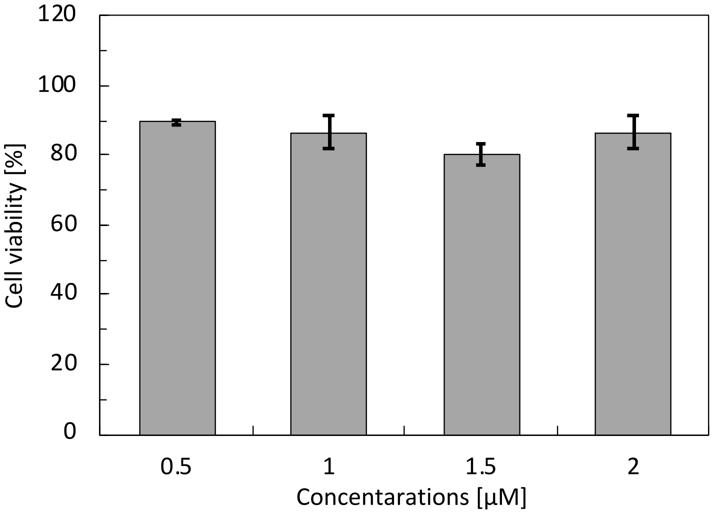

Figure S3 Cytotoxicity of minocycline against HeLa cells using MTT assay
Cytotoxicity of minocycline against HeLa cells using the MTT assay. The cell viability with PBS was normalized to 100%.

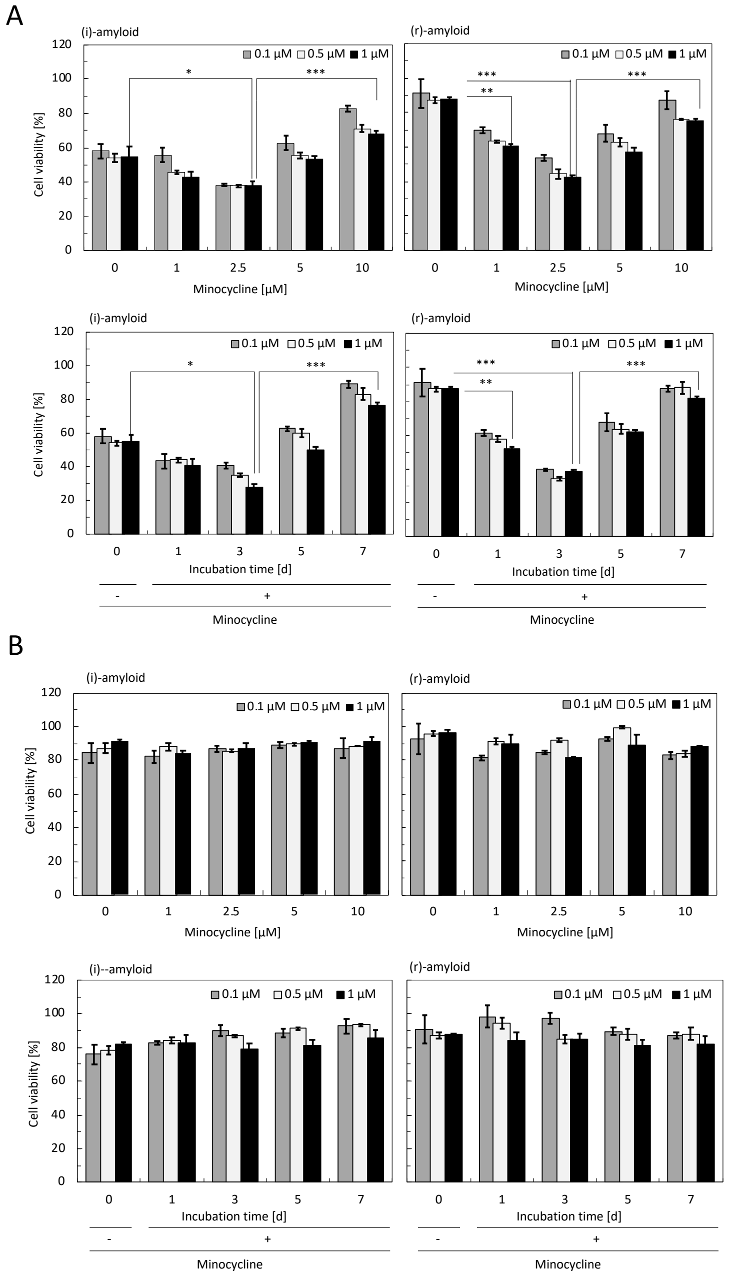

Figure S4 Cytotoxicity of the insulin amyloid samples in precipitate and supernatant using MTT assay against PC12 cells.

Cytotoxicity of the samples in the precipitate **(A)** and the supernatant **(B)** after centrifugation (15,000 rpm for 15 min) against PC12 cells using the MTT assay. The (i)-amyloid (left) and (r)-amyloid (right) samples were incubated with minocycline for 1 week at the indicated concentrations and centrifuged (upper) or with a fixed minocycline concentration (50 µM) for the indicated periods (lower). Samples incubated without minocycline for 7 days were shown as controls (0 µM and 0 day). All samples were quantified by BCA assay and were diluted to the same protein concentration: 0.1, 0.5, and 1 µM (gray, white, and black, respectively). The absorbance was normalized to 100% for PBS. (*P <0.05 , **P <0.01 , ***P <0.005)

A

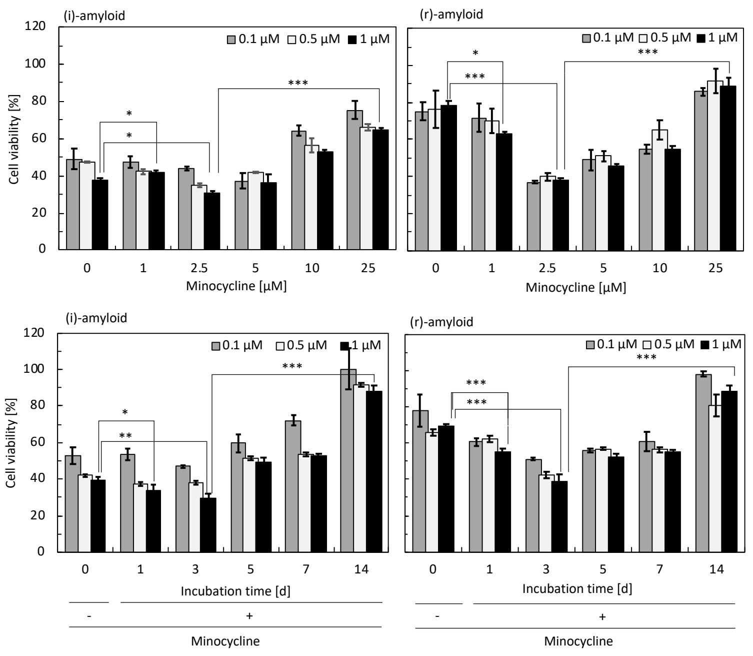

B

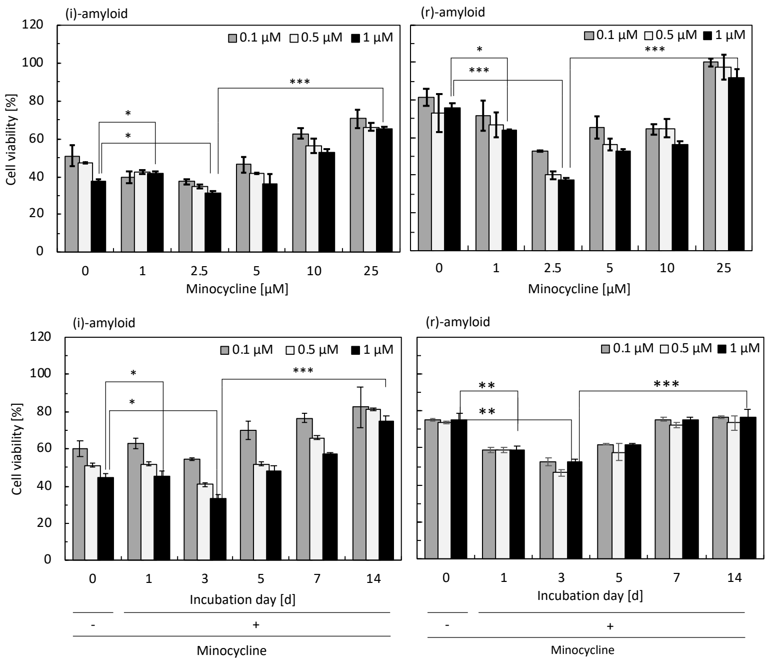

Figure S5 Cytotoxicity of the uncentrifuged insulin amyloid samples incubated with minocycline (uncentrifuged) using MTT assay against HeLa cells (A) and PC12 cells (B).

Cytotoxicity of the uncentrifuged samples against HeLa cells (A) and PC12 cells (B) using MTT assay. The (i)-amyloid (left) and (r)-amyloid (right) samples were incubated with minocycline for 1 week at the indicated concentrations and centrifuged (upper) or with a fixed minocycline concentration (50 µM) for the indicated periods (lower). Samples incubated without minocycline for 7 days were shown as controls (0 µM and 0 day). All samples were quantified by BCA assay and were diluted to the same protein concentration: 0.1, 0.5, and 1 µM (gray, white, and black, respectively). The absorbance was normalized to100% for PBS. (*P <0.05 , **P <0.01 , ***P <0.005)

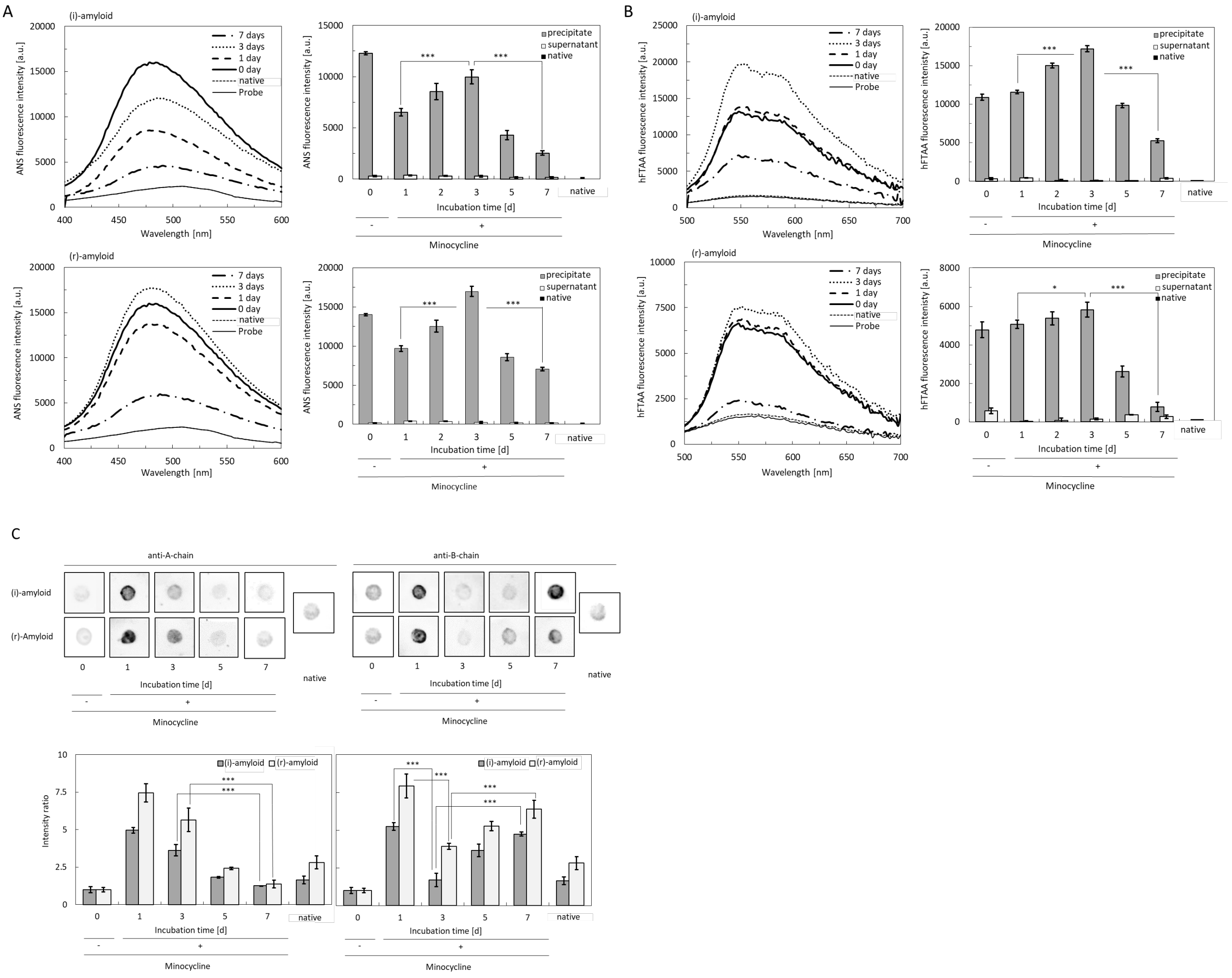

Figure S6 Structural changes of insulin amyloids incubated with minocycline dependent on incubation time.

The (i)-amyloid (upper) and (r)-amyloid (lower) samples were incubated with a fixed minocycline concentration (50 µM) for the indicated periods, and the precipitate and supernatant samples were evaluated by ANS **(A)** and hFTAA **(B)**. Left panels are the probe spectrum with: native insulin (thin-dotted line), probe (thin solid line), and samples at 0, 1, 3, and 7 days (thick solid, thick-dashed, thick dotted, and solid-dashed lines, respectively. The right panels are the peak fluorescence values at 480 nm (ANS) and at 560 nm (hFTAA): precipitate (gray), supernatant (white) and native insulin (black). The protein concentrations were adjusted to 5 µM. **(C)** Dot blot assay of the degradable amyloid samples in the precipitate by anti-insulin A chain and B chain antibody. The (i)-amyloid and (r)-amyloid samples were incubated with a fixed minocycline concentration (50 µM) for the indicated periods, and 5 µM precipitate samples after centrifugation were used. Graphs show the intensity analysis using the anti-A-chain antibody (left) and anti-B-chain antibody (right) by ImageJ: (i)-amyloid (gray) and (r)-amyloid (white). Intensities were normalized to 100 % for each untreated amyloid. (*P <0.05 , **P <0.01 , ***P <0.005)

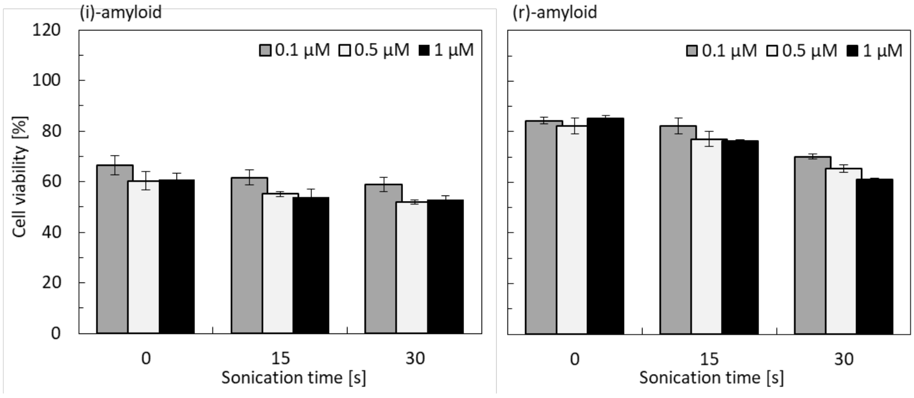

Figure S7 Cytotoxicity of the sonicated insulin amyloids using MTT assay against PC12 cells. Cytotoxicity of (i)-amyloid (left) and (r)-amyloid (right) sonicated for various times against PC12 cells, as evaluated by MTT assay. All samples were quantified by BCA assay and were diluted to the same protein concentration: 0.1, 0.5, and 1 μM (white, gray, and black, respectively). The absorbance was normalized to 100% for PBS.

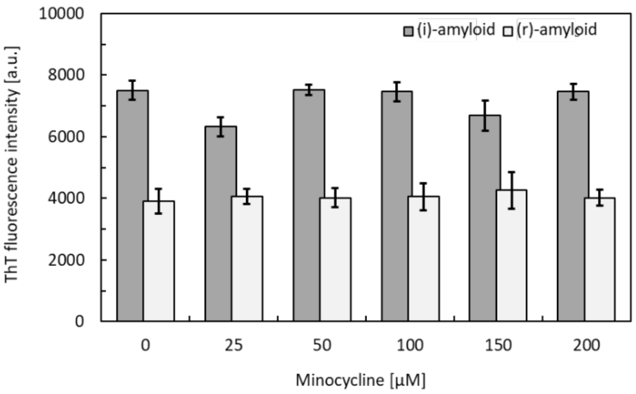

Figure S8 Effect of minocycline on the ThT assay of insulin amyloid.
ThT assay of (i)-amyloid (gray) and (r)-amyloid (white) treated with minocycline at various concentrations (0-200 μM).

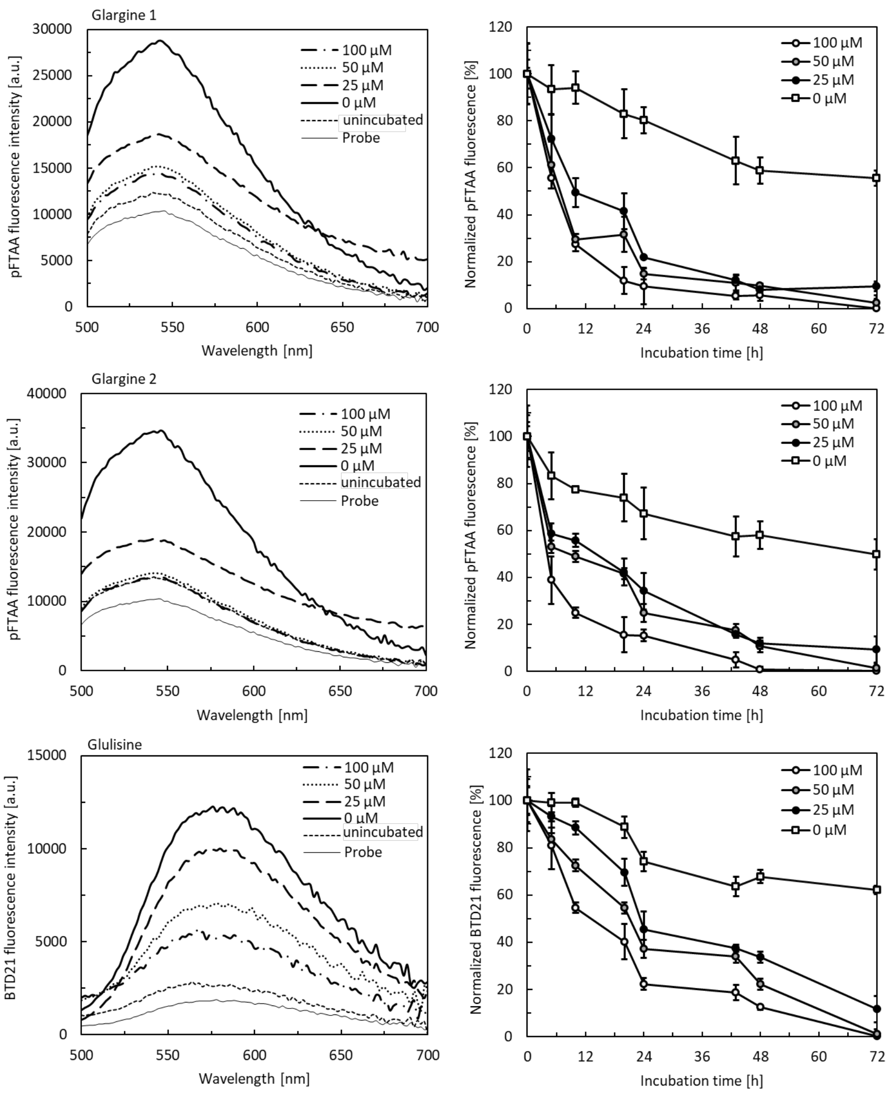

Figure S9 pFTAA and BTD21 assay of the insulin amyloids formed from insulin glargine 1, glargine 2 and glulisine incubated with minocycline.

pFTAA and BTD21 assay of glargine1, glargine2 and glulisine amyloid. Left images are the spectrum of pFTAA (glargine1 and glargine2) or BTD21 (glulisine) of insulin amyloid incubated for 72 hours: amyloid (thick solid line), 25, 50, and 100 µM minocycline (thick-dashed, thick-dotted, and solid-dashed lines, respectively;), unincubated insulin analogs (thin dotted line), and probe (thin solid line); right pictures are the plots of the intensities normalized for insulin amyloid as 100%: amyloid (white squares) and 25, 50, and 100 µM minocycline (black, gray, and white circles, respectively).

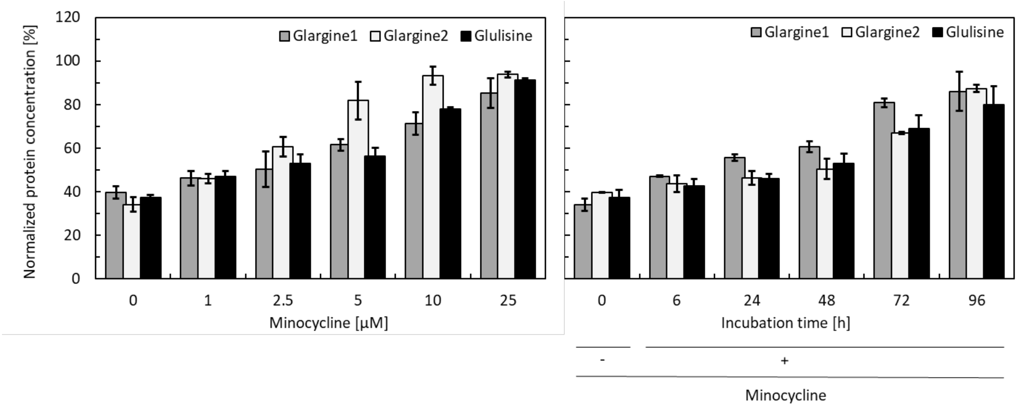

Figure S10 BCA assay for the quantification of degraded insulin analogs amyloid in the supernatant formed from insulin glargine 1, glargine 2 and glulisine.

BCA assay of supernatant samples to confirm the dependency of the degradation on minocycline concentration (upper) and incubation time (lower): glargine1 (gray), glargine2 (white), and glulisine (black). Values obtained from the 50 µM insulin treatment were normalized to 100%.

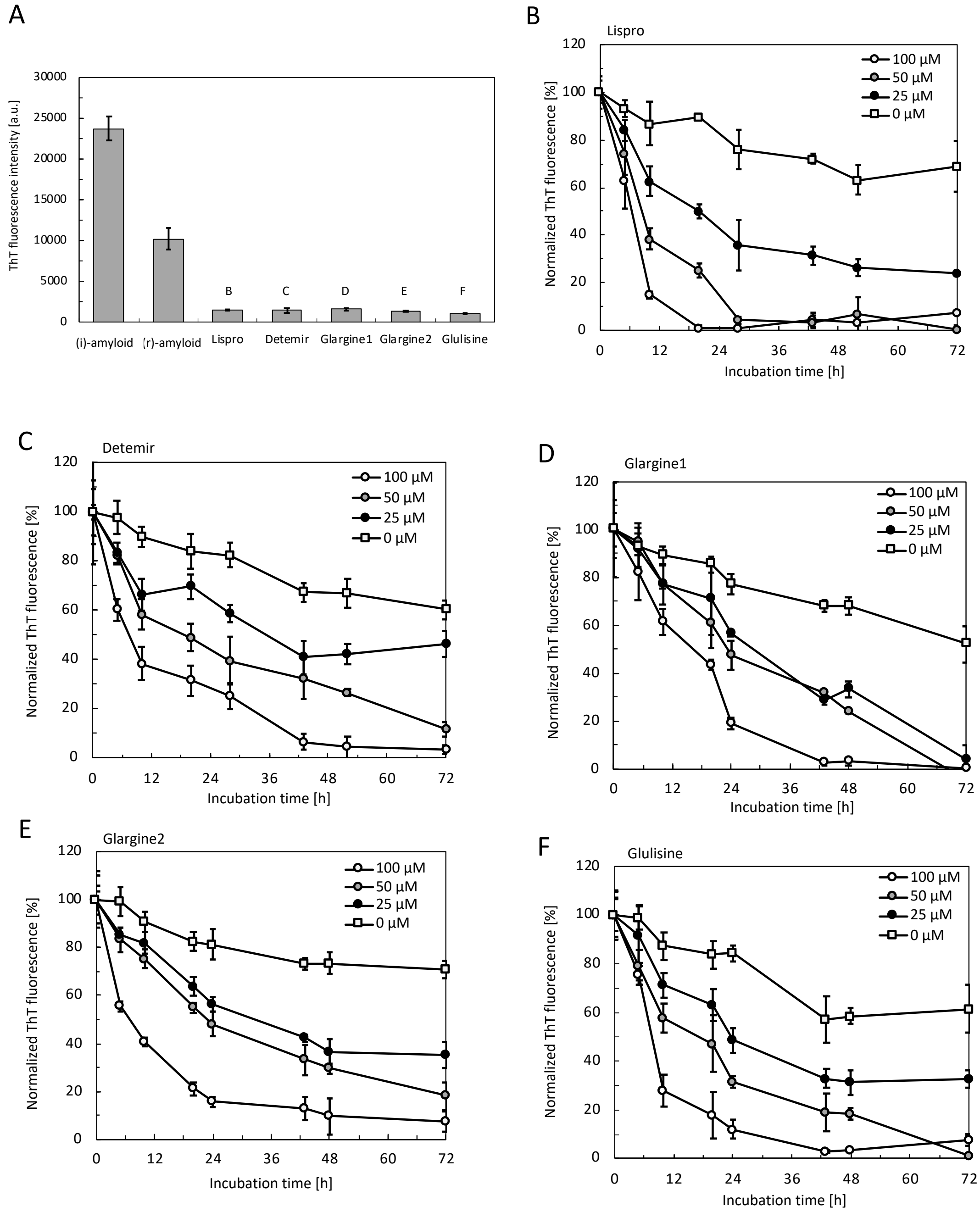

Figure S11 ThT assay of t the insulin amyloids formed from insulin lispro, detemir, glargine 1, glargine 2 and glulisine incubated with minocycline.

(A) ThT assay of insulin preparation. **(B)** lispro, **(C)** detemir, **(D)** glargine1, **(E)** glargine2 and **(F)** glulisine incubated in the presence of 0-100 µM minocycline: amyloid (white squares), and 25, 50, and 100 µM minocycline (black, gray, and white circles, respectively). ThT fluorescence was measured at 490 nm. Intensities were normalized for insulin amyloid as 100%. Intensities of the insulin amyloid at 0 h incubation time were normalized as 100%.

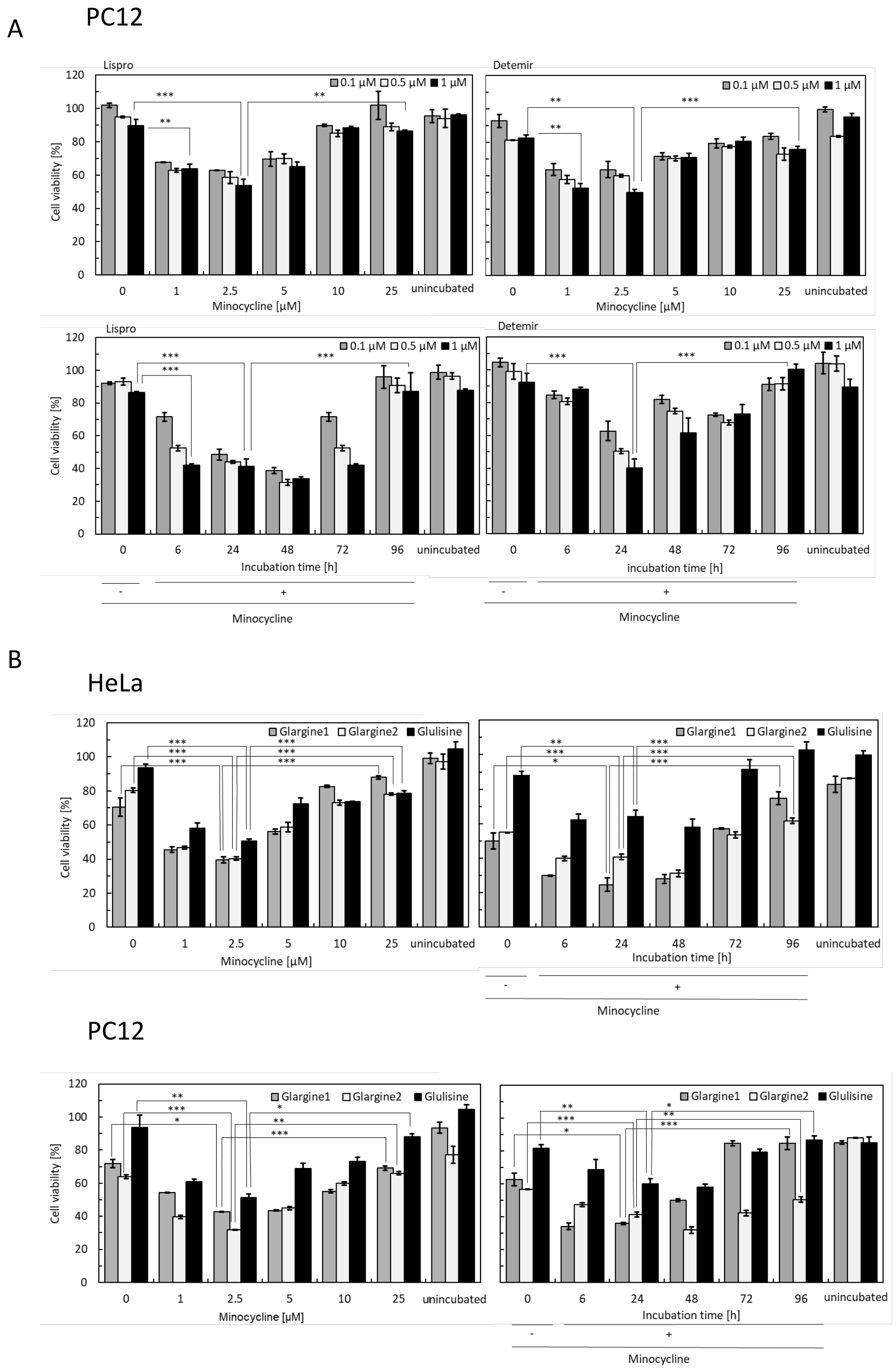

Figure S12 Cytotoxicity of the degraded amyloids formed by insulin preparations (lispro, detemir, glargine I, glargine II and glulisine)

(A) Cytotoxicity of lispro (left) and detemir (right) incubated with minocycline for 1 week at the indicated concentrations (upper) or with a fixed minocycline concentration (50 μM) for the indicated periods (lower) against PC12 cells. All samples were quantified by BCA assay and were diluted to the same protein concentration: 0.1, 0.5, and 1 μM (white, gray, and black, respectively). The absorbance

was normalized for PBS as 100%. (B) Cytotoxicity of galrgine1 (gray), galrgine2 (white), glulisine (black) incubated with minocycline for 1 week at the indicated concentrations (left) or with a fixed minocycline concentration (50 μM) for the indicated periods (right) against HeLa cells (upper) or PC12 cells (lower). All samples were quantified by BCA assay and were diluted to the same protein concentration (1 μM). (*$P < 0.05$, **$P < 0.01$, ***$P < 0.005$)

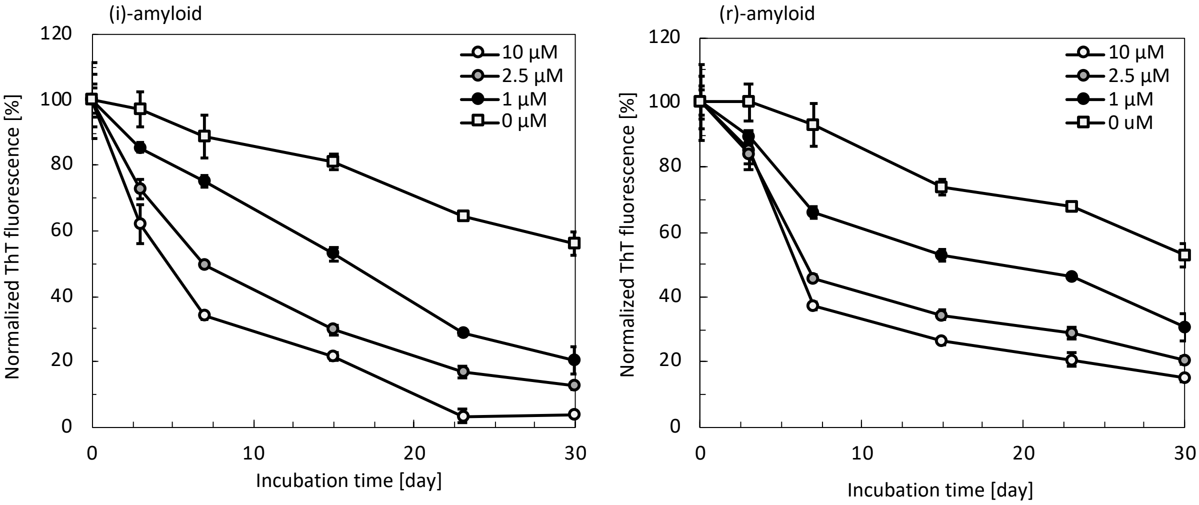

Figure S13 Degradation of insulin amyloids by minocycline at low concentrations

ThT assay of (i)-amyloid (left) and (r)-amyloid (right) incubated for an extended period of time in the presence of 0-10 µM minocycline. The intensities without incubation were normalized as 100%.
